# Native and prosthetic valve infective endocarditis complicated by rapidly progressive glomerulonephritis and its diagnostic challenges and therapeutic implications

**DOI:** 10.1002/ccr3.9054

**Published:** 2024-06-11

**Authors:** Yitagesu Getachew, Zerubabel Getahun, Getachew Wondafrash, Zemenay Asmare, Gashaw Solela, Beka Aberra, Merga Daba

**Affiliations:** ^1^ Cardiology Unit, Department of Internal Medicine Yekatit 12 Hospital Medical College Addis Ababa Ethiopia; ^2^ Department of Internal Medicine, School of Health Sciences Addis Ababa University Addis Ababa Ethiopia; ^3^ Division of Nephrology, Department of Internal Medicine Yekatit 12 Hospital Medical College Addis Ababa Ethiopia; ^4^ Department of Internal Medicine Yekatit 12 Hospital Medical College Addis Ababa Ethiopia; ^5^ Division of Nephrology, Department of Internal Medicine, School of Health Sciences Addis Ababa University Addis Ababa Ethiopia

**Keywords:** infective endocarditis, native valve endocarditis, prosthetic valve endocarditis, rapidly progressive glomerulonephritis

## Abstract

**Key Clinical Message:**

Concomitant native and prosthetic valve infective endocarditis (IE) is very rare, and both can rarely be complicated by rapidly progressive glomerulonephritis (RPGN). This diagnosis has therapeutic implications, as not all RPGN need immunosuppression therapy.

**Abstract:**

Native and prosthetic valve infective endocarditis (IE) may be rarely complicated by rapidly progressive glomerulonephritis (RPGN). The diagnosis of IE as a cause of RPGN may be missed, and patients may be subjected to inappropriate immune suppressive therapy. Moreover, IE involving multi‐valves has rarely been described, and there are only few case reports of simultaneous native and prosthetic valve endocarditis. Here, we present a case of 34‐year‐old female patient who has RPGN and whose initial workup missed IE. However, further workup revealed a diagnosis of native and prosthetic valve IE and our patient, who would have been subjected to inappropriate immune suppressive therapy, was treated with intravenous antibiotics alone and discharged with improvement.

## Graphical abstract

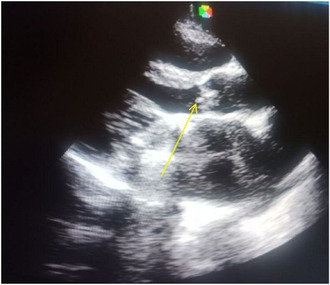

## 1 INTRODUCTION

Infective endocarditis (IE) is associated with many complications; these include cardiac, metastatic, neurologic, renal, musculoskeletal, and pulmonary complications. Renal complications of IE include glomerulonephritis (GN), renal infarction or abscess following septic embolization, and drug‐induced acute interstitial nephritis.^1^

Acute renal failure, defined as a serum creatinine of ≥2 mg/dL (177 mmol/L), has been described in up to one third of patients with infective endocarditis (IE).

IE can rarely be complicated by rapidly progressive glomerulonephritis (RPGN), and this poses diagnostic and therapeutic challenges. There are only a few case reports of IE associated with RPGN in the literature.

In a study of 595 IE‐related RPGN, the prevalence of IE‐related RPGN was found to be 3.4%.^2^ These studies have also found that it was difficult to differentiate these cases from other differentials, like vasculitis. In a retrospective review of 24 cases of IE associated with RPGN, all patients presented with fever and multisystem organ involvement. The involved organs include the heart, kidney, lung, skin, joint, spleen, lung, nervous system, and eye.^2^ All of these patients presented with rapid deterioration of renal function within 1 week to several months, along with glomerular hematuria and proteinuria. The mean peak serum creatinine was 6.7 mg/dL (range 3.0–16.1 mg/dL). The most commonly involved valves are the aortic and mitral valves. All of these patients were treated with intravenous antibiotics, and some of them required surgical and immune suppressive therapies.

Here we present the case of a 34‐year‐old known rheumatic heart disease patient for whom a prosthetic mitral valve replacement was done 14 years ago for severe mitral stenosis. The patient presented with flank pain, a decrease in urine amount, and a raised creatinine of 3 days duration. The initial workup with transthoracic echocardiography missed valvular vegetation. She was about to be started on immune suppressive therapy until repeat echocardiography revealed a 3 cm by 3 cm aortic and mitral valve vegetation. She was treated with intravenous antibiotics alone and diuretics, and she did not require immune suppressive therapy. She was discharged with improvement, and follow‐up transthoracic echocardiography revealed resolution of the vegetation.

## 2 CASE PRESENTATION

A 34‐year‐old female patient who was on follow‐up for chronic rheumatic valvular heart disease, for whom a prosthetic mitral valve replacement was done 13 years ago for severe mitral stenosis, came to our hospital OPD with the complaint of flank pain of 3 days duration. The pain was dull and aching in type and involved both her flank areas. Associated with this, she also had reddish discoloration of the urine, a decrease in urine volume, and lower extremity swelling of the same duration. On further inquiry, she admitted that she had a low‐grade intermittent fever, loss of appetite, and easy fatigability, which started during this illness period. She was taking anticoagulation and antibiotic prophylaxis. Other than the symptoms mentioned above, she had no orthopnea, PND, chest pain, or chronic medical illnesses like HIV, diabetes mellitus, or hypertension.

On physical examination, she was afebrile; her blood pressure was systolic 110 mmHg and diastolic 80 mmHg; her pulse rate was 78 bpm; her respiratory rate was 18 breaths per minute; her morning axillary temperature was 36.7°C; and her oxygen saturation was 92% with atmospheric oxygen. Cardiovascular examination revealed a flat JVP and a Grade IV holosystolic murmur that is best heard at the apex. Further examination of the genitourinary system showed bilateral flank tenderness, and muscoskeleton examination showed grade II pitting edema involving both extremities.

**FIGURE 1:**
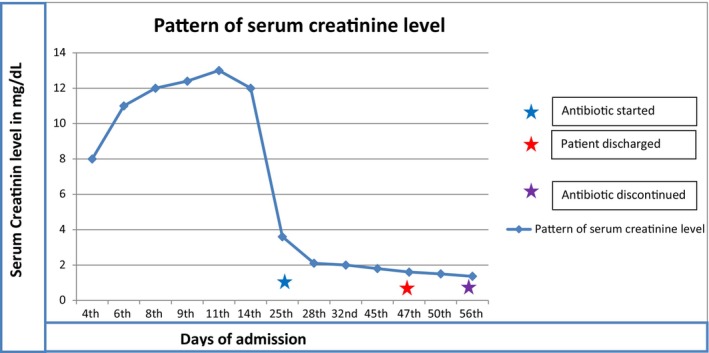
Pattern of serum creatinine level.

**FIGURE 2:**
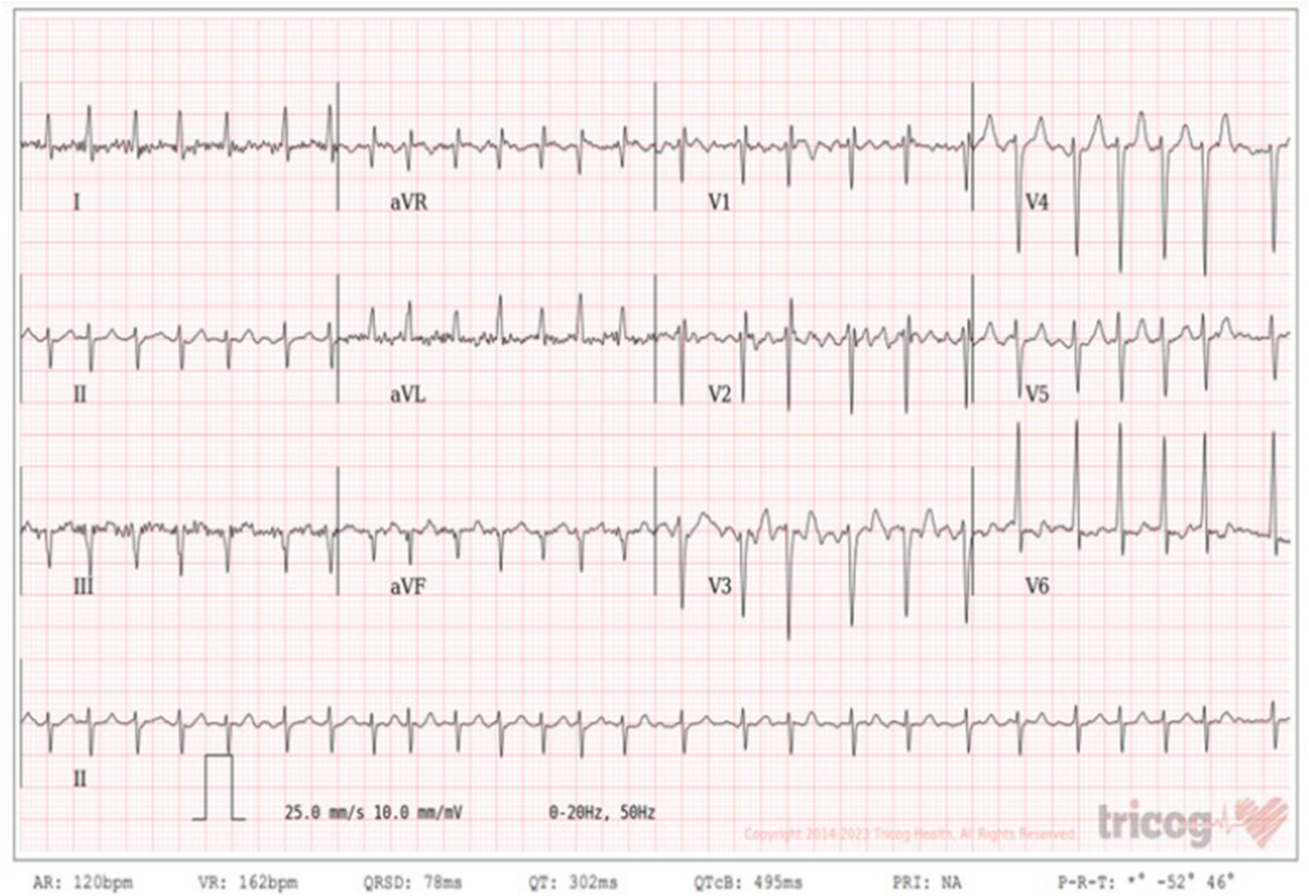
The ECG showed features of atrial fibrillation with a fast ventricular response of 165 bpm.

We investigated her with a complete blood count, which showed a WBC of 25,000, a neutrophil percentage of 91%, and hemoglobin and platelet counts that were within normal (Table 1, trend of CBC). Her coagulation profiles were INR: 2.8, PT: 33.6 s, and PTT: 55 s. She had an elevated acute phase reactant (ESR: 68 mm/h). Her serum creatinine was 8.5 mg/dL; her baseline serum creatinine was normal 2 weeks prior to our hospital visit (Figure 1, trend of serum creatinine). Urine analysis showed proteinuria and many red blood cells, and the 24‐hour urine protein determination was 244 mg. Serology tests like ANA, hepatitis B surface antigen, hepatitis C antibody, and HIV tests were all negative. She was further investigated with a chest X‐ray, which was normal, and an ECG showed features of atrial fibrillation with a fast ventricular response of 165 bpm, and the rate was controlled up on discharge (Figure 2). With the initial suspicion of IE, we investigated her with a blood culture and echocardiography. The blood culture showed no microbial growth after 7 days.

**TABLE 1:** CBC trend.

Days of admission		1st day	7th day	25th day	28th day
CBC	WBC	25k	17k	14k	7k
	N	91%	90%	85%	74%
	L	3.5%	3.9%	5.3%	19%
	Hgb	16.1	19	12.9	13
	MCV	105.2	106	100	89
	PLT	129	69	169	359

Initial transthoracic echocardiography was normal. As transesophageal echocardiography was not available in our set up, we repeated transthoracic echocardiography after a week, and it showed a 3 cm by 3 cm of vegetation on the aortic and prosthetic mitral valves, severe aortic stenosis, moderate aortic regurgitation, moderate mitral stenosis, severe tricuspid regurgitation, and mild pulmonary hypertension. Mild biventricular systolic dysfunction with an ejection fraction of 45%.

She was admitted to our hospital, and a diagnosis of rapidly progressive glomerulonephritis (RPGN) was made, and we planned to give her methylprednisolone pulse therapy. However, the repeated echocardiography showed vegetation over both the native valve (aortic) and the prosthetic valve (mitral valve), as described above. Later, a diagnosis of native and prosthetic IE complicated by RPGN was made, and the plan to give pulse therapy was canceled. For this diagnosis, we treated our patient with intravenous antibiotics, vancomycin, and cefepime for a duration of 6 weeks and diuretic therapy. Our patient didn't require any sessions of hemodialysis. After 2 weeks of these treatments, flank pain and lower extremity edema subsided. Serum creatinine recovered, and the patient was discharged with improvement. She was seen on follow‐up, and she was in good condition.

**FIGURE 3:**
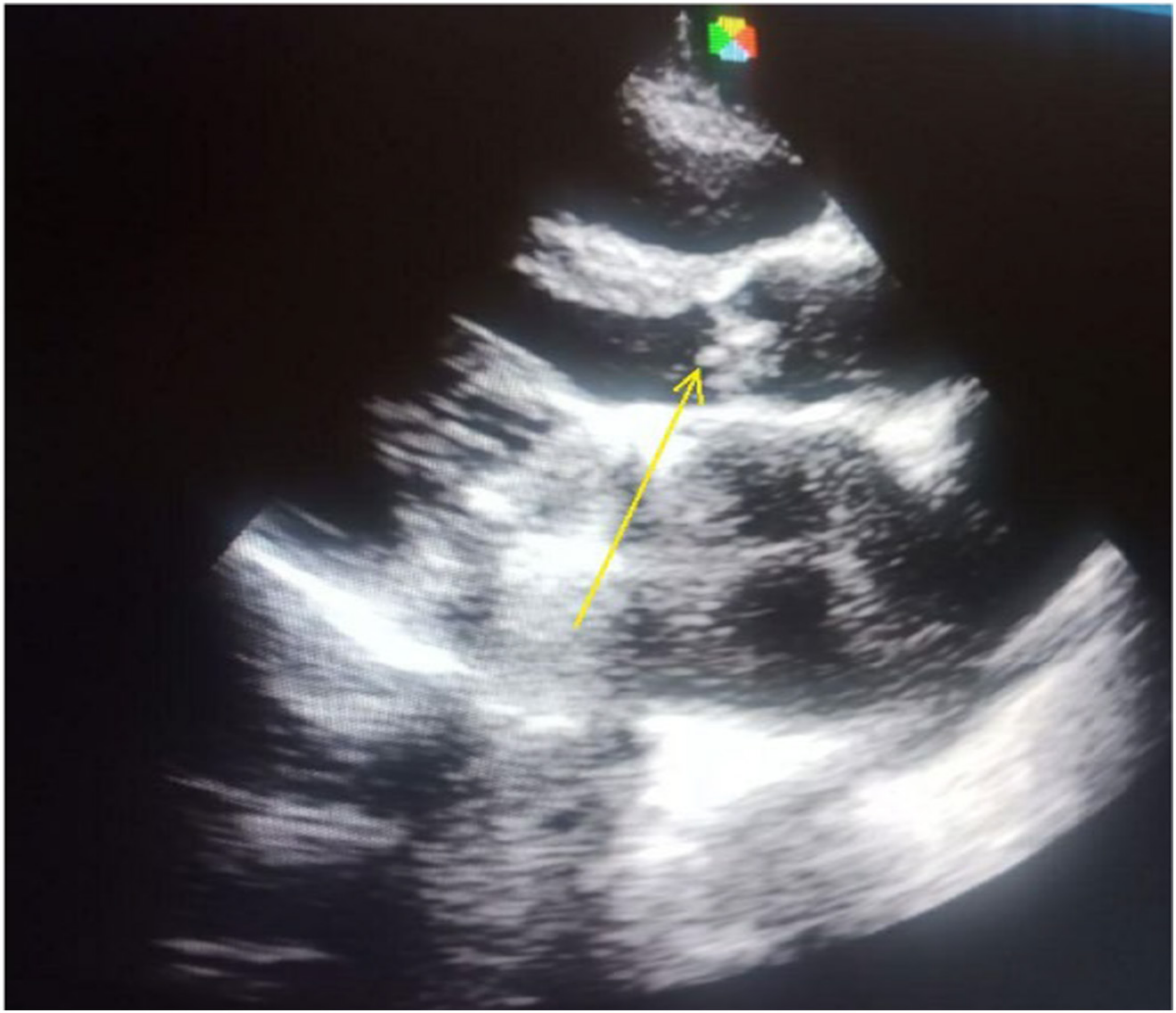
Echocardiography image post‐treatment: arrow head shows aortic valve vegetation (the prosthetic mitral valve vegetation has disappeared).

The follow‐up transthoracic echocardiography showed aortic vegetation, which has decreased in size; otherwise, prosthetic mitral valve vegetation was not seen (Figure 3), and the follow‐up serum creatinine was normal.

## 3 DISCUSSION

IE is an infection of the endocardial surface of the heart, and it can be an infection of one or more heart valves or of the intracardiac devices. It is a rare disease with an estimated yearly incidence of 3–10 cases per 10,000 people.^3^

The term native valve endocarditis refers to a cardiac infection that involves the leaflets of the valves, the endocardial surface, chordae tendinae, congenital defects, and anastomosis sites. Prosthetic valve endocarditis is a microbial infection of parts of a prosthetic valve or reconstructed native heart valve.^4^

Patients with IE can develop several forms of kidney disease. These include bacterial infection‐related immune complex‐mediated GN, renal infarction from septic emboli, and renal cortical necrosis.

RPGN is a clinical syndrome manifested by features of glomerular disease in the urinalysis and by progressive loss of kidney function over a comparatively short period of time (days, weeks, or a few months). The treatment we give to patients with IE can also lead to kidney injury.^5^, ^6^, ^7^

Diagnoses of IE as a cause of RPGN have therapeutic implications. Most patients with RPGN need immune suppressive therapy. However, patients whose RPGN is caused by IE may be successfully treated with antibiotics alone and may not need immune suppressive therapy. Our patient has clinical and laboratory features suggestive of RPGN, and we planned to give immune suppressive therapy. It is not uncommon for transthoracic echocardiography to miss valvular vegetation. The initial echocardiography did not show valvular vegetation, but the repeated one showed three small‐sized aortic and prosthetic mitral valve vegetations. Our patient fulfills diagnostic clinical criteria for both native and prosthetic valve IE,^8^ and a diagnosis of native and prosthetic valve IE complicated with RPGN was made.

Infective endocarditis involving multiple valves has rarely been described, and there are only a few case reports of simultaneous native and prosthetic valve endocarditis.^9^ Multivalvular and simultaneous native (aortic) and prosthetic (mitral) valve involvement is what makes our patient's presentation unique.

Studies have shown that patients with IE‐associated RPGN can be treated with intravenous antibiotics, and some of these patients may need immune suppressive therapy, particularly when kidney function does not improve after antibiotic therapy.^2^ We treated our patient with intravenous antibiotics, and she showed clinical improvement, and serum creatinine showed recovery over 2 weeks.

Our patient would have been subjected to immune suppressive therapy if the diagnosis of IE had been missed.

## 4 CONCLUSION

Native and prosthetic valves IE may be rarely complicated by RPGN. The diagnosis of IE may be missed, especially if the vegetation is small, as observed in our patient. Detection of IE as a cause of RPGN may eliminate the need for immune suppressive therapy, as observed in our patient. Our case illustrates that not all RPGN require immunosuppressive therapy. So, it is important to have a high index of suspicion of IE as a cause of RPGN in appropriate clinical conditions.

## AUTHOR CONTRIBUTIONS

**Yitagesu Getachew:** Conceptualization; investigation; validation; writing – review and editing. **Zerubabel Getahun:** Investigation; writing – original draft. **Getachew Wondafrash:** Conceptualization; supervision; validation. **Zemenay Asmare:** Formal analysis; writing – original draft. **Gashaw Solela:** Supervision; writing – review and editing. **Beka Aberra:** Supervision; validation. **Merga Daba:** Writing – original draft; writing – review and editing.

## FUNDING INFORMATION

No funding was used in this case report.

## CONFLICT OF INTEREST STATEMENT

The authors authors have no conflict of interest.

## ETHICS STATEMENT

The authors' institution does not require ethical approval for publication of single case report.

## CONSENT

The patient provided written informed consent for the publication of details including, history, physical findings, laboratory reports, and imaging. Written informed consent was obtained from the patient to publish this report in accordance with the journal's patient consent policy.

## Data Availability

The data that support the findings of this case report are available from the corresponding author upon reasonable request.
